# Nanomaterials in Drug Delivery: Strengths and Opportunities in Medicine

**DOI:** 10.3390/molecules29112584

**Published:** 2024-05-31

**Authors:** Chinedu O. Egwu, Chinyere Aloke, Kenneth T. Onwe, Chukwunalu Igbudu Umoke, Joseph Nwafor, Robert A. Eyo, Jennifer Adaeze Chukwu, Godswill O. Ufebe, Jennifer Ladokun, David Tersoo Audu, Anthony O. Agwu, David Chukwu Obasi, Chukwuemeka O. Okoro

**Affiliations:** 1 Medical Research Council, London School of Hygiene and Tropical Medicine, Banjul 220, The Gambia; 2 Medical Biochemistry Department, College of Medicine, Alex-Ekwueme Federal University Ndufu-Alike, P.M.B. 1010, Ikwo 482131, Nigeria; alokec2002@yahoo.com (C.A.); roberteyo@gmail.com (R.A.E.); odomeroufebe@gmail.com (G.O.U.); akatonyagwu@gmail.com (A.O.A.); 3 Protein Structure-Function and Research Unit, School of Molecular and Cell Biology, Faculty of Science, University of the Witwatersrand, Braamfontein, Johannesburg 2050, South Africa; 4 Anatomy Department, College of Medicine, Alex-Ekwueme Federal University Ndufu-Alike, P.M.B. 1010, Ikwo 482131, Nigeria; onwe.kenneth@funai.edu.ng (K.T.O.); chukwunalucc@gmail.com (C.I.U.); jnwafor49@gmail.com (J.N.); 5 World Health Organization, United Nations House Plot 617/618 Central Area District, P.M.B. 2861, Abuja 900211, Nigeria; gennytimah@gmail.com; 6 Society for Family Health, 20 Omotayo Ojo Street, Allen, Ikeja 100246, Nigeria; jenniferladokun@gmail.com; 7 UNICEF Sokoto Field Office, 2 Rahamaniyya Street, Off Sama Road, Sokoto 840224, Nigeria; aududt@yahoo.co.uk; 8 Department of Medical Biochemistry, David Umahi Federal University of Health Sciences, Uburu 491105, Nigeria; davisbassey4may@gmail.com (D.C.O.); organizzer4real@gmail.com (C.O.O.)

**Keywords:** nanotechnology, cancer, infectious diseases, treatment, targeted-delivery, controlled-release, low-toxicity, high-efficacy

## Abstract

There is a myriad of diseases that plague the world ranging from infectious, cancer and other chronic diseases with varying interventions. However, the dynamism of causative agents of infectious diseases and incessant mutations accompanying other forms of chronic diseases like cancer, have worsened the treatment outcomes. These factors often lead to treatment failure via different drug resistance mechanisms. More so, the cost of developing newer drugs is huge. This underscores the need for a paradigm shift in the drug delivery approach in order to achieve desired treatment outcomes. There is intensified research in nanomedicine, which has shown promises in improving the therapeutic outcome of drugs at preclinical stages with increased efficacy and reduced toxicity. Regardless of the huge benefits of nanotechnology in drug delivery, challenges such as regulatory approval, scalability, cost implication and potential toxicity must be addressed via streamlining of regulatory hurdles and increased research funding. In conclusion, the idea of nanotechnology in drug delivery holds immense promise for optimizing therapeutic outcomes. This work presents opportunities to revolutionize treatment strategies, providing expert opinions on translating the huge amount of research in nanomedicine into clinical benefits for patients with resistant infections and cancer.

## 1. Introduction

Nanotechnology is the branch of science and technology that deals with the manipulation and control of materials, structures, and devices at the nanoscale, typically ranging from 1 to 100 nanometers [1]. It involves understanding and utilizing unique properties and phenomena that occur at the nanoscale to create innovative applications across various fields, such as medicine, electronics, energy, and materials science [2,3]. Nanotechnology has been applied in medicine in several ways which include diagnosis, drug delivery and/or actuation purposes in a living organism [4].

Drug delivery entails the movement of a drug to its target site to exert its pharmacological effect. Drugs could be delivered to their targets primarily through the major routes of drug administration which include: peroral, intravenous, intramuscular, subcutaneous, sublingual and rectal routes. Drugs delivered for treatment of diseases via these routes are currently facing a lot of challenges that lead to treatment failure due to diverse reasons, which may include, but are not limited to, efflux of drugs from target sites, drug degradation during delivery and drug toxicities. To enhance drug delivery at target sites and reduce toxicity, newer approaches in drug delivery are imperative. Reduced access to target sites and retention time at these sites have been highlighted as being among the causes of treatment failure. Nanotechnology is a vital tool that has the potential to address these shortfalls in the conventional method of drug delivery and has seen several useful applications in research and medicine in treatment and diagnosis. The drugs loaded in NPs interact with them either chemically via electrostatic and/or hydrophobic interactions, or physically dissolved into the NP through encapsulation. NPs classified as nano-drug delivery systems possess the capacity to enhance drug stability and water solubility, prolong drug circulation, boost the rate of uptake by target cells or tissues, and decrease enzyme degradation. These features ultimately lead to an enhancement in drug safety and efficacy [5]. As a precise, efficient, and controllable method for intracellular drug delivery, NPs present unique advantages in the diagnosis and treatment of cardiovascular diseases (CVDs) and other diseases. They effectively address challenges related to targeting, localized drug delivery, controlled and sustained release, and toxicity reduction. This work aims to X-ray the strengths and opportunities that abound with the deployment of nanotechnology in drug delivery and the challenges encountered in the use of this technology in medicine. This work will also proffer solutions on how to improve the use of the technology in clinics for the benefits of patients.

## 2. Drug Delivery Methods

The ultimate aim of drug administration is for the drug to reach the target site and perform its pharmacological role. Drug delivery is the process of providing a pharmaceutical substance in order to obtain therapeutic effects in people or animals [6]. To ensure that this happens, drugs are designed and packaged to enhance delivery to the target sites. Drug delivery has become a focal point in pharmaceutical and medical sciences in order to improve therapeutic outcomes. Drug delivery is a field of medicine and pharmaceuticals that focuses on the controlled and targeted administration of therapeutic substances to specific areas in the body. The primary goal of drug delivery systems is to enhance the efficacy and safety of medications while minimizing side effects and improving patient compliance [7]. Drug delivery can be performed through several approaches as highlighted below.

### 2.1. Traditional Drug Delivery System (TDDS)

In the past, most drugs were administered in conventional forms such as tablets, capsules, and injections. These formulations release the drug systemically into the bloodstream, leading to a broader distribution throughout the body. While effective, they may result in side effects and may not precisely target the intended site of action or diseased cells [7]. The most common methods of the TDDS include the oral, topical, trans-mucosal (nasal, buccal, sublingual, vaginal, ocular, rectal), parenteral and inhalation routes [8]. These conventional approaches have certain limitations that may reduce their efficacy. These limitations include, but are not limited to, drug instability, risk of displacement, uncontrolled release, side effects, slow absorption and enzymatic deterioration [4].

### 2.2. Advanced Drug Delivery System (ADDS)

Due to the shortfalls of the TDDS, innovations on improving drug delivery have been a constant process. The ADDS is geared towards circumventing the traditional routes and delivers the drug directly to the target sites in the right amounts and when due [9]. Poor bioavailability and changes in plasma drug level characterize traditional drug delivery modalities (tablets, capsules, syrups, ointments, etc.) [9]. The ADDS improves efficacy and reduces toxicity via targeted drug delivery and controlled drug release, among other mechanisms. Nanotechnology provides a formidable tool in the ADDS.

The ADDS reduces dosage and frequency of doses while minimizing negative effects. Surface functionalization of nanoparticles is another important design aspect and is often accomplished by bioconjugation or passive adsorption of molecules onto the nanoparticle surface. By functionalizing nanoparticle surfaces with ligands that enhance drug binding, suppress immune response, or provide targeting/controlled release capabilities, both a greater efficacy and lower toxicity are achieved.

Advancements in drug delivery have revolutionized the pharmaceutical industry, enabling more effective treatments for a wide range of diseases. Targeted and controlled drug delivery systems hold promises for personalized medicine and improved patient outcomes. However, the development of such systems requires rigorous testing, safety assessments, and regulatory approvals before they can be widely used in clinics.

The conventional medications delivered via traditional approaches are not necessarily produced using the best formulation for each product. For products containing proteins or nucleic acids to increase their effectiveness and safeguard them from unintended degradation, a more novel kind of delivery system is needed [10].

#### 2.2.1. Targeted Drug Delivery

ADDSs aim to improve drug targeting by delivering medications directly to the site of disease or action, raising the concentration of the drug in such target areas relatively higher than other non-target areas. This can be achieved by using various approaches, such as encapsulating drugs in nanoparticles, which can then be designed to accumulate in specific tissues or cells [11]. NPs in targeted drug delivery prolong and localize drugs’ actions, enhancing the bioavailability, biodistribution and accumulation of therapeutics specifically in diseased areas [12], minimizing contact with healthy ones. This reduces toxicity and improves efficacy outcome.

**Figure 1:**
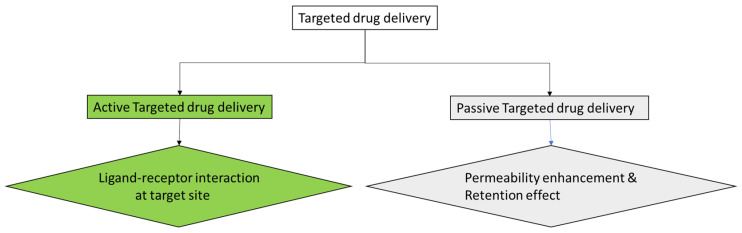
Mechanism of targeted drug delivery. Adapted from [13].

Targeted drug delivery can be classified into two categories: active and passive targeted delivery (Figure 1). While active targeted drug delivery is based on ligands interaction to receptors, the passive approach relies on the enhanced permeability and retention effect (EPR effect) [13].

In active targeted drug delivery, targeting is based on ligands interaction to receptors. The drug-nanoparticles have specific ligands that have the ability to recognize specific receptors, which are concentrated or relatively higher in the diseased cells in comparison to the healthy ones. This ensures that the drug-NP accumulates only in the diseased area. For instance, cancer cells receptors are often different from normal tissues. The overexpression of receptors on the cancer cells helps them to meet their nutritional needs in order to carry on their hypermetabolism [14].

For passive targeted-delivery, the leaky vasculature and impaired lymphatic drainage are exploited to make for selective and enhanced delivery in diseased cells. In diseased cells like tumors, leaky blood vessels are formed, which become more permeable than normal cells. The leaky nature may not allow drugs to accumulate due to the size of the drugs. Secondly, the absence of normal lymphatic drainage in tumor contributes to the NPs retention. Therefore, coupling of the drugs to NPs can increase their permeation and retention in such diseased cells [15]. This ultimately improves drug bioavailability and prolongs the duration of action of the drug at target sites causing minimal side effects.

#### 2.2.2. Controlled Drug Release

**Figure 2:**
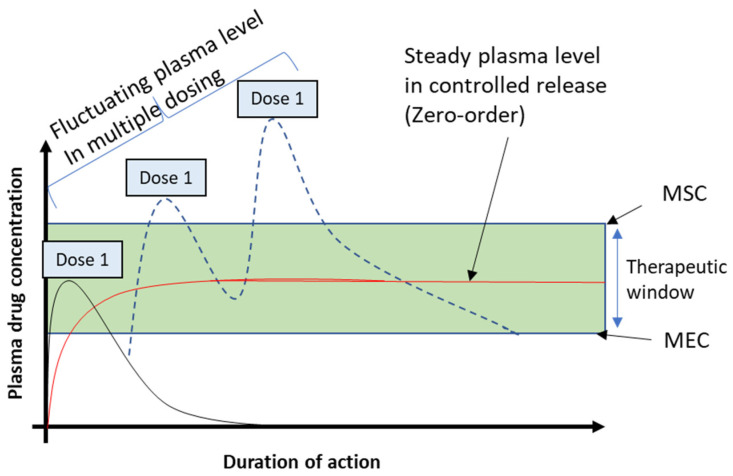
Mechanism of controlled drug release. Adapted from [17].

Controlled release is an important application of nanotechnology in drug delivery that ensures the precise and gradual release of drugs from nanoparticles or other carriers over an extended period. This approach ensures a sustained and steady supply of the medication at the target site, resulting in optimized therapeutic effects and improved patient compliance [16]. Drugs are effective at doses that lie between the minimum effective concentration (MEC) and the maximum safe concentration (MSC) [17]. This is called the therapeutic window (Figure 2). Conventionally, trying to achieve this effective concentration may come at a cost. High doses of a drug that can meet the therapeutic requirements may also be toxic (higher than MSC), while reducing the doses to avoid the toxicity will reduce the efficacy of such drugs. To avoid the risk of toxicity at high doses and poor efficacy at low doses, some drugs have been administered at intermittent periods of the day, say, 2–4 times daily. This complicates the treatment regimen and adversely affects patients’ compliance. This underscores the need for intermittent release of drugs based on the therapeutic systemic requirements. The controlled drug release provides a zero-order drug release profile where the concentration released remains the same over time (Figure 2).

The drugs are encapsulated in NPs, which are then released intermittently in response to certain stimuli, which could be internal or external such as temperature, pH, ionic strength, sound, and electric or magnetic fields [16]. The basic mechanisms that control the release of the drug molecules from the nanoparticles’ layers are osmosis, diffusion, chemical degradation, swelling and dissolution; however, diffusion is the dominant mechanism, where the drug is released into the environment from the polymer matrix without eroding the hydrogel NPs. The release of the drug is affected by several factors including the physicochemical properties of the active agent, structural characteristics of matrices, and the release medium [18].

#### 2.2.3. Improvement in Drug Stability

Drug stability entails the ability of the drug in a particular formulation (tablet, capsules, injectables etc.) to retain its physical, chemical, therapeutic, microbial and toxicological properties during the time of storage and usage by the patient [19]. The stability of a drug is important for the drug to reach the target sites for their pharmacological activities. More so, the durability/stability during storage is dependent on the constituents of the drug. It is therefore imperative that the stability of drugs during design is considered. For this reason, drug stability can broadly be classified into five categories: chemical, physical, microbiological, therapeutic, and toxicological stability. Degraded drugs may not only be less effective for an intended disease condition, but may also pose severe adverse events such toxicities and predisposing the patients to cancer. It becomes necessary to protect the stability of the drugs.

The stability of a drug is affected by factors such as temperature, light, pH, duration of storage and very importantly, the packaging materials. To increase the stability of drugs, NPs have become handy. The size and surface properties of NPs have been explored to increase their stability [20]. While the drug stability is improved with NP packaging, it is equally important for the drugs to be releasable when they reach their target sites, otherwise the ultimate aim of improved therapeutic outcome will be defeated.

#### 2.2.4. Improvement of Pharmacokinetic Properties of Drugs

The pharmacokinetics of drugs entail the absorption, distribution, biotransformation and excretion. Each of these processes affect the effect of a drug and the therapeutic outcome. Through the coupling of drugs to NPs, favorable pharmacokinetic properties are improved such as better absorption, enhanced distribution to target sites, improved metabolism and excretion. The unique small size and large surface area allow drug-NPs to have increased solubility; hence, enhanced bioavailability. More so, their ability to cross the blood brain barrier (BBB), enter the pulmonary system and be absorbed through the tight junctions of endothelial cells of the skin improves their pharmacokinetic properties and therapeutic outcomes [20].

For some drugs that show low bioavailability, slowing down their metabolism and excretion can increase their therapeutic window and outcomes. Drugs entrapped in NPs show reduced liver metabolism and renal clearance, resulting to prolonged blood circulation with an increased chance of accumulation in the target tissue [21].

## 3. Nanoparticles Used in Drug Delivery

Nano-carriers such as liposomes, micelles, dendritic macromolecules, quantum dots, carbon nanotubes and metal-based NPs have been widely used in treatments especially for cancer.

### 3.1. Liposomes

**Figure 3:**
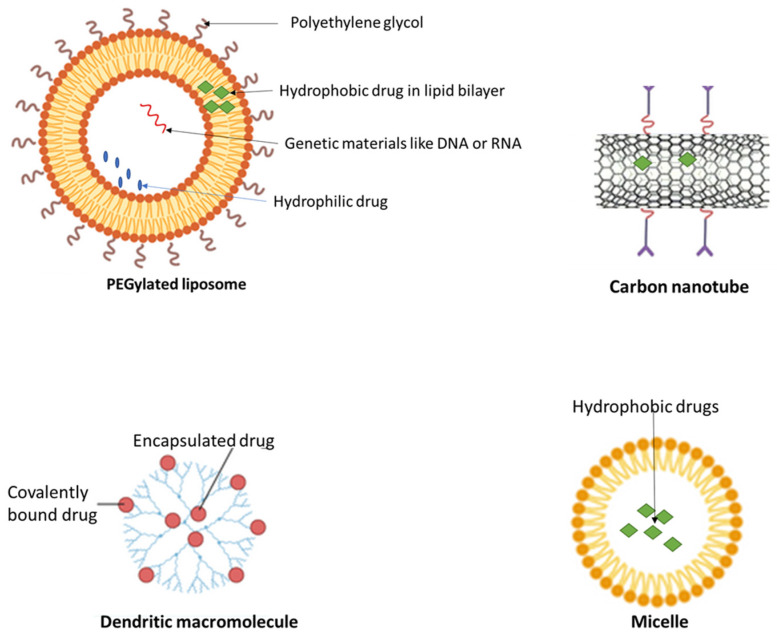
Common nanoparticles used in drug delivery. Figure templates adapted from BioRender.

Liposomes are spherical vesicles composed of synthetic or natural phospholipids that self-assemble in aqueous solutions in sizes ranging from tens of nanometers to micrometers [2]. The resulting vesicle, which has an aqueous core surrounded by a hydrophobic membrane, can be loaded with a wide variety of hydrophobic or hydrophilic molecules for therapeutic purposes (Figure 3). As drug delivery systems, liposomes display exceptional qualities such good biocompatibility and safety, prolonging the half-life of the medication, regulating the release of drug molecules, and shielding the encapsulated compounds from physiological degradation [22,23].

The release of a drug from liposomes depends on the liposome composition, pH, osmotic gradient, and the surrounding environment [24]. Moreover, a prolonged residence period lengthens the duration of such particles’ actions, while reducing their number that could cause toxicity. Adsorption, fusion, endocytosis, and lipid transfer are among ways that liposomes and cells might interact. Liposomal formulations have a wide range of therapeutic examples, including anticancer medications [8], neurotransmitters (serotonin) [25], antibiotics [26], anti-inflammatory medications [27], and antirheumatic medications [28]. Some of the liposome-delivered drugs that have been approved for use are as shown in Table 1, while others are still at different phases of clinical trial (Table 2).

Liposomes can encapsulate both lipophilic and hydrophilic drugs. The major drawbacks of liposomes are leakage and fusion of encapsulated drug, poor solubility, low stability and short half-life [29]. The modification of liposomes by using biopolymers or use of post-processing techniques like drying to generate dry liposomes improve the stability of liposomes [30].

**Table 1:** Nanoparticles-delivered drugs in the market.

Drug	Trade Name	Administration Route	Approved Indication	Reference(s)
**Liposomes**				
Amphotericin B	AmBisome (Astellas)	Intravenous	Fungal infections	[31]
Amphotericin B	Fungizone	Intravenous	Fungal infections	[31]
Daunorubicin	DaunoXome (Galen)	Intravenous	Leukemia	[32]
Doxorubicin	Doxil (Baxter Hlthcare Corp)	Intravenous	Combination therapy with cyclophosphamide in metastatic breast cancer	[33]
Verteporfin	Visudyne (Valeant Luxembourg)	Intravenous	Age-related molecular degeneration, pathological myopia, ocular histoplasmosis	[34]
Cytarabine	DepoCyt© (Pacira Pharms Inc.)	Spinal	Neoplastic meningitis and lymphomatous meningitis	[35]
Morphine sulphate	DepoDur	Epidural	Pain management	[36]
Moderna vaccine	Moderna	Intravenous	COVID-19	[37]
Pfizer-BioNTech	Pfizer-BioNTech	Intravenous	COVID-19	[37]
Mifamurtide	Mepact (Takeda)	intravenous	Osteosarcoma, a form of bone cancer	[38]
Recombinant varicella-zoster virus glycoprotein E	Shingrix	Intramuscular	Against shingles and post-herpetic neuralgia	[39]
**Micelles**				
Docetaxel	Taxotere (Sanofi Aventis)	Intravenous	Antineoplastic	[40]
Estradiol	Estrasorb™ (Novavax)	Dermal	Menopausal therapy	[41]
**Dendritic macromolecules**				
VivaGel	VivaGel R© BV (Starpharma)	Dermal	Treatment and symptomatic relief of bacterial vaginosis	[42]
**Quantum dots**				
Aprepitant	Emend (Merck)	Oral	Vomiting agent	[43,44]
Megestrol acetate	MegaceES (Endo Pharms Inc.)	Oral	Anorexia	[45]
Dexamethylphenidate HCl	Focalin XR/(Novartis)	Oral	Mental stimulant	[46]
Tizanidine HCl	Zanaflex (Covis)	Oral	Muscle relaxant	[47]
Nabilone	Cesamet (Bausch)	Oral	Antiemetic	[48]
Naproxen sodium	Naprelan (Almatica)	Oral	Anti-inflammation	[49]
Griseofulvin	Gris-PEG (Novartis)	Oral	Fungal infection	[48]

**Table 2:** Selected Nanoparticles-delivered drugs in development.

Active Ingredient	Drug Candidate ID	Phase of Development	Indication	Reference(s)
Liposome				
Cisplatin	SPI-77	III	Different forms of Cancer	[50]
Cisplatin	Lipoplatin	III	Different forms of Cancer	[50]
Amphotericin	Ambisome	III	Fungal Infection	[50]
Micelles				
Cisplatin	Nanoplatin	III	Different forms of Cancer	[50]
Paclitaxel	NK-105	III	Breast cancer	[51]
Doxorubicin	SP1049-C	III	Cancer	[52]

The majority of the nanodrugs in development are at Phase I (33%) followed by those in Phase II (21%), where the majority of the drug candidates target cancer (53%), followed by infectious diseases (14%) [53].

### 3.2. Micelles

Micelles are self-assembled microstructures formed by surfactants in an aqueous system, forming a hydrophilic exterior and hydrophobic core (Figure 3), and are usually <50 nm in diameter [54]. While the hydrophobic core can house and deliver hydrophobic drugs, the hydrophilic surfaces offer protection to the structure, preventing it from easy dissolution in the physiological system [55]. For many years, micelles have been investigated as drug delivery systems. Through the improved permeability and retention effect (EPR), their usage may result in significant drug accumulation at the target site. The solubility of hydrophobic drugs is also enhanced when loaded in micelles. More so, the drug can be loaded for distribution at the desired locations, which decreases drug loss and adverse effects while increasing drug bioavailability at the needed diseased areas [55]. They can be used to deliver hydrophobic drugs such as paclitaxel, tamoxifen, porphyrin, camptothecin and vitamin K3 [54]. Some micelle-delivered drugs that are already approved for clinical use are shown in Table 1.

Micelles are good at delivering water-insoluble drugs [56]. However, poor solubility of small-sized micelles, poor loading capacity, and poor physical stability are big issues [57]. These can be addressed by covalent or non-covalent crosslinking to stabilize the structure. The stability can also be improved via other non-crosslinking approaches like tweaking the hydrophilic/hydrophobic block ratios of the micelles and elevating the crystallinity of hydrophobic segments [57].

### 3.3. Dendritic Macromolecules

Dendritic macromolecules were first developed in 1978 by Buhleier et al. under the name cascade polymers [58]. They are hyperbranched polymers with a three-dimensional structure (Figure 3). The three-dimensional architecture of dendrimers can incorporate a variety of biologically active agents to form biologically active conjugates [59]. Dendritic molecules can interact with the drugs through several means, including covalent bonding, chemical conjugation system and/or physical interactions like encapsulation. Dendritic macromolecules have been used in delivering several anticancer agents such as 5-fluorouracil, cisplatin, doxorubicin, Methotrexate and Paclitaxel [60].

Dendrimers are also used in gene delivery [61,62]. For instance, Polyamidoamine (PAMAM) dendrimers modified with gold nanorods can be deployed to deliver short hairpin RNA, where they enhance gene transfection efficacy and this can be applied in killing cancer cells. Only few dendrimer-based products have been approved for clinical use; however, many are at different phases of clinical trial as shown in Table 1. The polyvalency of dendritic molecules allows them to encapsulate multiple drugs while allowing the controlled addition of targeting onto the nanocarriers. More so, they offer high physical stability to the drugs [63]. Their major drawbacks are their low hydrosolubility and high non-specific toxicity, which can be addressed by modifying the cationic dendrimers to become neutral or completely modify them to anions [64,65].

### 3.4. Quantum Dots

Quantum dots (QDs) are semiconductors, (also known as nanocrystals) that are a few nanometres in size and have optical and electronic properties, which can be exploited for drug delivery and diagnosis. These particles are composed of an inorganic core semiconductor, such as Cadmium selenide (CdSe), and an organic-coated aqueous shell, such as Zinc sulphide (ZnS) [48]. QDs are seen as efficient fluorescent labels used in a drug delivery system for monitoring the drug biotransformation in the body. The composition of the core determines the fluorescence, while the aqueous outer surface determines the type of drug to be conjugated. Drugs such as 5-fluorouracil, doxorubicin, and paclitaxel loaded with QDs showed remarkable improvements in therapeutic outcomes [66]. Some drugs delivered in QDs for clinical use are as shown in Table 1. The optical property of QDs makes them unique for medical imaging too; however, the presence of heavy metals makes them toxic [67].

### 3.5. Carbon Nanotubes

Carbon nanotubes (CNTs) are formed by a layer of carbon atoms that are bonded together in a hexagonal mesh, wrapped into a cylinder of approximately 2.5–100 nm in diameter [68] (Figure 3). They can be functionalized by adding proteins or DNA, which serve as therapeutic agents. CNTs can access cells easily, delivering drugs directly to the cytoplasm or nucleus. CNTs can also complement the efforts of drugs by providing free radicals scavenging functions, acting as antioxidants [69].

Even though CNTs are yet to be tested in humans, they have shown promising signs in the delivery of several drug and genetic materials. CNTs have been used as carriers for some anticancer agents like docetaxel, doxorubicin, methotrexate, paclitaxel, and gemcitabine [68]. This is especially important as CNTs have appropriate porosity and poses low toxicity [70].

### 3.6. Metal-Based Nanoparticles

Metal-based NPs contain metals as a component of the NP. Metal-based NPs are classified into four categories: metallic, bimetallic, metal oxide and magnetic NPs [71]. The metallic NPs are pure forms of metal-based NPs, which are also called metal NPs (e.g., silver, gold, copper and zinc [72]. The metal NPs can be synthesized from their metal precursors, and modified with various chemical functional groups, which allow them to be conjugated with antibodies, ligands, and drugs of choice. The bimetallic NPs are composed of two metals, exploiting the good qualities of each element. Common examples are iron–cobalt (Fe-Co), Gold-silver (Au-Ag) and iron-platinum (Fe-Pt). They have a better ability to penetrate and release bioactive substances in cells in comparison to monometallic NPs; hence, they are exploitable in the treatment of bacterial infections [73]. For the metallic oxide, the main examples are titanium dioxide (TiO_2_) cerium dioxide (CeO_2_), silica (SiO_2_), iron oxide (Fe_2_O_3_) and zinc oxide (ZnO). These metallic oxide NPs have found useful application in nutrient supplementation and cancer treatment as exemplified by Fe_2_O_3_ in supplementation in anaemia and advanced cancer treatments [74]. The magnetic NPs (MNPs) are those that have magnetic core covered by either organic (e.g., liposomes) or inorganic (e.g., silica) moieties along with therapeutic agents. They have been exploited in theranostics of cancer via magnetic resonance imaging (MRI) and treatment. The metals commonly used for MNP designs are iron, cobalt, nickel, and manganese [75].

Metal-based NPs have the advantages of high specific surface area and small particle size, increasing their wide application in the biological field. Because of their non-specific bacterial toxicity mechanism, they have been positioned to treat resistant infections [76]. One of the drawbacks of metal-based NPs is their cytotoxicity, which is attributed to the reactivity of the metals leading to bulking. Hence, the use of more stable metals (less reactive) like gold is advocated to overcome this challenge [71].

## 4. Applications of Nanotechnology in Drug Delivery

By tweaking the size, surface characteristics and material used, the NPs can be developed into smart systems, which can encapsulate therapeutic and imaging agents [20]. These modifications in the NPs can enhance delivery of the drugs at specific sites, minimizing side effects from such drugs. Nanotechnology has therefore found useful applications in the treatment of diseases especially where the conventional deliveries have failed [77].

### 4.1. Treatment of Resistant Infectious Diseases

Treatment of infectious diseases is increasingly difficult due to evolutionary dynamism of the causative agents and rising drug abuses.

#### 4.1.1. Addressing Antimalarial Drug Resistance

Malaria is among the leading causes of death in the tropical and subtropical regions of the world. Many antimalarials are in use for the treatment of malaria. However, their relevance in the treatment wanes a few years after the drug introduction into the market. This is mainly due to treatment failures arising from antimalarial drug resistance. Resistance development has molecular mechanisms, affecting several metabolic pathways. The main genes affected include, but are not limited to, *Pfcrt*, *mdr* and *k13* genes in malaria parasites [78]. This has led to the incessant search for newer antimalarials, which is usually cost intensive and tasking. Nanotechnology has been projected as a promising tool in addressing antimalarial drug resistance. Each class of antimalarial has its strength and weakness. The use of nanotechnology is geared towards making up for the weaknesses of these antimalarials. For instance, artemisinin and its derivatives are the gold standard recommended by the World Health Organization because of rapid action and proven efficacy [79]; however, due its short half-life, the benefits therein are reducing. Delivery of artemisinins on nanoparticles can release the drugs intermittently, overcoming the short half-life, resulting in improved efficacy. Repackaging the drugs on nanocarriers improves their pharmacokinetic profiles, which increases their therapeutic outcome [20,21]. Many studies on the use of nanotechnology in delivering antimalarials have shown promising results; however, only a few of these studies have reached the clinical stages [80]. In the adoption of this technology, the active components remain in the conventional drugs such as artemisinin and chloroquine, combined with a delivery system [29,81].

#### 4.1.2. Treatment of Bacterial Multi-Drug Resistance (MDR)

**Figure 4:**
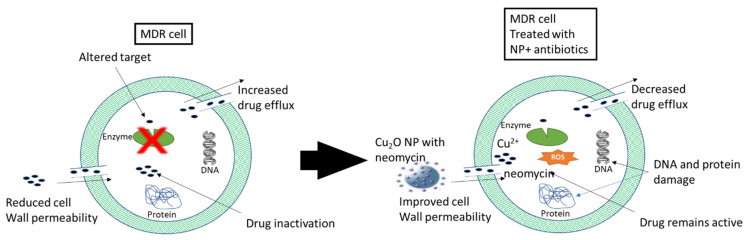
Overcoming bacterial resistance via NP drug delivery. Adapted from [84,85]. Antibiotic drug resistance is characterized by increased drug efflux, reduced cell wall permeability and altered targets, which are addressed by coupling with NP, which reduces the drug efflux, improves cell wall permeability causing increases in drug concentration and therapeutic outcome.

The use of antibiotics has become increasingly indiscriminate and characterized by gross abuse and misuse. This leads to the development of multi-drug resistance (acquired non-susceptibility to at least one agent in three or more antimicrobial categories), which worsens control of bacterial infection. Multi-drug resistance (MDR) develops as a result of efflux of drugs from target sites, antibiotic enzymatic deactivation, reduced cell wall permeability etc. (Figure 4). This calls for new drug development or a change in strategies to combat the threat posed by MDR globally. Nanoparticles have shown effective antimicrobial activity against MDR bacteria, such as *Acinetobacter baumanii*, *Pseudomonas aeruginosa*, *Klebsiella pneumoniae*, *Mycobacterium tuberculosis*, vancomycin resistant *enterococci, methicillin-resistant Staphylococcus aureus* etc. NPs alone have promising results in killing bacteria [82,83]. The paradigm shift in medicine is the combination of NPs and antibiotics for effective treatment of MDR cases [84]. The NPs tend to undo the mechanisms of MDR via cell wall and membrane collapse, reactive oxygen species (ROS) generation, binding to and damaging intracellular components and/or destruction of biofilm architecture [85].

The use of antibacterial agents in combination with NPs improves their efficacy against Gram-positive and Gram-negative bacteria, in addition to drug-resistant bacteria. For instance, the combination of silver NPs with bacitracin, ciprofloxacin, tetracycline, and cefixime against *P. aeruginosa*, *E. coli*, *S. aureus*, and *Candida albicans* showed synergy [86]. In a similar manner, the combination of Zinc Oxide NPs with Vancomycin, and ampicillin had a synergistic effect against MDR *Enterococcus feacium* [87]. In another study, Cu_2_O-NP with aminoglycoside antibiotics (e.g., neomycin) showed enhanced efficacy against *Escherichia coli* [88].

### 4.2. Cancer Therapy

**Figure 5:**
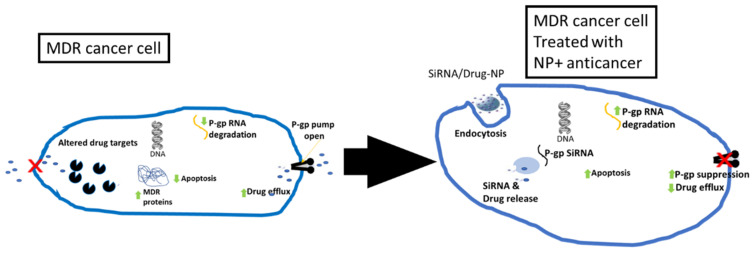
Overcoming cancer drug resistance via NP-drug delivery. Adapted from [95,96]. NP in delivering cancer drugs helps to overcome the drug efflux in cancer cells by taking drugs into cells by endocytosis and P-gp suppression.

According to the World Health Organization (WHO), cancer is among the leading causes of death, which was responsible for one in every six deaths globally in 2020 [89]. Cancer develops at a particular part of the body and spreads to other parts in a process termed metastasis. Early detection and treatment are very important in the fight against cancer. Nanotechnology has found a place in cancer diagnosis and treatment and has led to several promising results in cancer therapy [90]. For this reason, attention has been focused on consolidating the gains of nanotechnology in cancer therapy. Anticancer drug resistance develops due to several factors, among which is efflux of a drug from the target site, preventing pharmacologically relevant doses from accumulating [91]. The role of nanotechnology is to efficiently deliver and accumulate these anticancer agents to their target sites [92] (Figure 5). This improves treatment outcome.

Several studies have highlighted the improved therapeutic outcome in cancer management when the anticancer agents are delivered in NPs. NP-based drug delivery systems are linked to improved pharmacokinetics, biocompatibility, tumor targeting, and stability of drugs that tend to reverse drug resistance associated with cancer chemotherapy [93]. This also leads to reduced toxicities associated with cancer chemotherapy.

A recent study revealed the effectiveness of overcoming drug resistance in lung cancer therapy by combining miR495 and doxorubicin into a cancer cell membrane-coated silica nanoparticle, the results of which indicated that miR495 effectively down-regulated P-gp expression in multi-drug resistant cancer cells [94]. In a review by Babu et al., co-delivery of SiRNA and other anticancer agents (which is usually challenging ordinarily) showed significantly improved outcome using NPs in managing resistant cancer [95]. In the review by Babu et al., many anticancer agents and SiRNA combinations co-delivered by NPs were considered and they all showed promising results through P-glycoprotein (P-gp) suppression, increased apoptosis and reduced drug efflux. In another study, Bai et al. demonstrated that NP-mediated drug delivery to the tumor neovasculature overcome P-gp-expressing multi-drug resistant, showing better efficacy when compared to P-gp and other chemotherapy combination [96].

### 4.3. Treatment of Cardiovascular Diseases

Cardiovascular diseases (CVDs) are the leading cause of death globally. They are diseases that affect the heart and the blood vessels such as hyperlipidemia, hypertension, myocardial infarction, stroke and thrombosis. These conditions are chronic and have to be managed for a long time. It becomes imperative to factor in efficacy, safety and compliance in the management of CVDs. Due to polypharmacy that is common in some chronic conditions, drug administration as a single dose with sustained release is often sought. The use of nanotechnology can deliver these drugs in a more effective manner either via targeted delivery or controlled release in order to increase efficacy and compliance and also to reduce side effects. Several efforts have been made in improving the pharmacokinetic behavior of drugs used in managing CVDs. For instance, the use of carvedilol loaded in solid lipidic nanoparticles (SLN) improved the bioavailability of the carvedilol, an antihypertensive whose solubility is poor [97]. Similarly, Kumar et al. demonstrated that delivering nitrendipine in SLN improves its bioavailability by four to five times [98]. These modifications in pharmacokinetics improves the therapeutic outcomes of the drugs.

### 4.4. Administration of Nutraceuticals

Nutraceuticals are food or food components that have medicinal and health values, including the prevention and treatment of disease. They may show antioxidant, anti-inflammatory, antimicrobial, and antineoplastic actions [99]. The efficiency of bioactive compounds in drugs, foods, and food supplements is assessed by means of concepts like bioavailability, bioaccessibility, bioactivity, bioconversion, and bioequivalence [100]. The composition of nutraceuticals and their size affect their liberation in the GIT and absorption across the lipid membranes, thereby reducing their bioavailability. The unique qualities of NPs such as small size, superficial electrical charge and high surface/volume ratio, make them the best approach in addressing the bioavailability loss in nutraceuticals [101]. The NPs improve the bioavailability of nutraceuticals by protecting them from degradation, improving their solubility in aqueous environments and increasing their intestinal permeation and transcellular delivery [102]. Various NPs have been developed (e.g., lipid-based, surfactant-based, and biopolymeric NPs) to uniquely deliver both lipophilic and hydrophilic nutraceuticals in order to improve their bioavailability [103].

### 4.5. Gene Therapy

Treatment of some diseases via chemotherapy has been hampered due to several factors including drug resistance linked to genetic modifications. Delivery of appropriate genetic materials to correct the issues have been adopted. However, the uptake of such genetic materials into the cells may be challenging. Gene delivery using NP is a promising technology that can revolutionize gene therapy. Gene delivery is a specialized form of drug delivery that involves introducing genetic material into cells to treat genetic disorders or modify cellular behavior [104]. NPs have been explored in the delivery of genetic materials for breast cancer treatment as it improves the circulation time, bioavailability, reduces the immune system-based recognition chances, and delivers the gene regulator accurately [105]. Gene delivery systems could be composed of lipids, polymers, polypeptides, graphene-family nanomaterials, inorganic materials (e.g., gold NPs) or a combination of these materials [106]. Short interfering RNAs (SiRNAs) and plasmid DNA can be delivered by polyester-based and lipid NPs, respectively, [107,108] while messenger RNAs are also delivered by lipid NPs, which is exemplified in the popular mRNA COVID-19 vaccines [109].

## 5. Challenges of Nanotechnology

For the design and effective use of nanotechnology in drug delivery, key considerations must be made. Some of these considerations include: Good knowledge of their components and their interactions, identification of key characteristics and their relation to performance, ability to replicate key characteristics under manufacturing conditions, ease of manufacture in a sterile form, ability to reach and accumulate in the desired site of action by overcoming the restrictive biological barriers and ease of storage and administration [110]. Some of the challenges that currently face the use of nanotechnology are as follows:

### 5.1. Toxicity and Biocompatibility

Some NPs may possess inherent toxic properties, which can be dependent on their composition, size, and surface characteristics. The intention in the design of drug-NPs is to produce little or no unfavorable response when interacting with the host, that is, being biocompatible. Protein-based NPs show mild toxicities ranging from hepatoxicity, nephrotoxicity and cardiotoxicity, hypersensitivity reactions, aggregation and instability, while lipid-based NPs may cause cardiopulmonary distress, anaphylactoid reactions and some allergic reactions just like protein-based NPs. However, for metal-based NPs, they affect mainly the growth and developmental systems, causing depression, genotoxicity, reproductive toxicity, DNA damage and cellular inflammations [111]. Understanding and mitigating potential toxic effects are vital for safe clinical applications. Nanoparticles can have unique properties due to their small size, which may lead to unexpected biological interactions [112]. Some NPs have been found to induce cellular damage or inflammation, posing potential health risks. Researchers must thoroughly assess the toxicity of NPs to ensure they do not harm healthy cells or tissues in the body before considering them for therapy. The toxicity and biocompatibility of NPs depend largely on the composition and to some extent, their stability. It is therefore important to develop NP-drugs that are stable and will not degrade during administration or storage.

### 5.2. Cost of Production

The cost of production in the context of nanotechnology-based drug delivery systems refers to the expenses incurred during the development, manufacturing, and distribution of these systems. Several factors contribute to the overall cost of production, which may include research and development, cost of materials, manufacturing process and/or quality control. The huge cost involvement is sometimes discouraging and does not allow the patients to enjoy the full benefits therein. While nanotechnology-based drug delivery systems offer significant therapeutic potentials, their production costs can be relatively high, impacting their accessibility and affordability for patients. Ongoing research and advancements in manufacturing techniques may help to reduce production costs over time, making these innovative systems more accessible to a broader population. The cost involvement may limit the promises provided by nanotechnology in medicine [113].

### 5.3. Regulatory Challenges

Nanomedicine is an upcoming area that has a lot of promises in medicine. It is heralded by a lot of regulatory challenges. Regulatory challenges are significant barriers that can affect the development, approval, and commercialization of nanotechnology-based drug delivery systems. The major regulatory challenges are lack of global standardization of nomenclature, test methods or characterization [114]. NPs often exhibit complex physicochemical properties, making their characterization and standardization challenging. Regulatory agencies may require standardized testing methods and well-defined criteria for quality control and reproducibility. Because nanomedicine is an upcoming area in medicine, safety concerns are always there, especially the unknown long-term effects. Regulatory agencies require thorough toxicological and safety assessments, which can be time-consuming and costly.

To circumvent these challenges, efforts are ongoing from the regulatory agencies to develop guidelines and frameworks specific to nanotechnology. Collaborations by researchers and companies at different stages of the drug development is necessary to fine tune the areas of concern in the course of development. Some of regulatory agencies involved in nanomedicine regulation are the US Food and Drug Administration, European Medicines Agency (EMA) and other agencies from Asia and America. While the agencies promote research and innovations, ensuring the safety of the innovations when already in the market through pharmacovigilance is central to their mandate. This is intended to protect the end users from any form of harm that could arise from the long-term use of nanomedicine. In 2007, several agencies like the EMA, the FDA and others from Japan, Canada and Australia came together to develop ways on how to define the characteristics of medicines based on nanotechnology [115]. This was aimed at increasing uniformity in characterization of nanomedicine and a concerted effort in regulation.

### 5.4. Composition of the Drug and Nanoparticles

The drugs carried in NPs usually constitute less than 5% of the entire composition. To ensure that the percentage of the NP is kept low and NP toxicity is avoided, the surface-area-to-volume ratio of NPs can be altered to allow for more ligand binding to the surface. This reduces the number of NPs required for the delivery of a given quantity of the drug, increasing the efficiency of delivery [116]. During the preparation, sometimes, the drug content may be lower than required, which will reduce its efficacy and overall efficiency. There may also be a scenario where the NPs are higher than required and pose toxicity to the users. A careful balance must be sought in composing the drug-NPs. Generally, drugs delivered in NPs can be composed in different forms of NPs such as polymeric NPs, inorganic NPs, viral NPs, lipid-based NPs, and nanoparticle albumin-bound (nab) technology. The choice of a NP for drug delivery depends on several factors including the target environment and desired therapeutic outcome [117]. Moreover, the biodegradability of the NPs should be considered as the biodegradable ones ensure sustained drug release within the target site over a period of time [118], and at the same time, prevent the risk of toxicity from the NP after drug delivery. The NPs should have high pore volume, narrow pore diameter distribution, and high surface area to enhance the encapsulation of the drugs and ensure intermittent release at the target site [119].

More so, due to poor composition, the drugs may be released prematurely in a process termed “burst release”. A portion of the drugs are simply adsorbed on the surface of the NP. Consequently, a significant fraction of the drug will be released before reaching the pharmacological target in the body, leading to low therapeutic outcome and higher toxicity [15]. The mechanisms through which NPs improve the delivery of drugs (targeted delivery, controlled release, improved pharmacokinetic ability and increased stability lie largely on the composition of the entire materials. The size and surface properties of nanomaterials have to be fully explored and exploited to optimize composition and delivery of the drugs [20].

### 5.5. Poor Biodistribution

Biodistribution deals with the manner in which NPs access different tissues in the body such as the liver, the lungs and the spleen. The distribution of NPs into these tissues is determined by some physico-chemical properties of the NPs such as size, surface charge and coating [120,121]. For instance, magnetic NPs (MNPs) > 100 nm in size largely accumulate in the liver, spleen and the lungs, while smaller sized MNPs (10 nm) are filtered by the kidney and almost eliminated via renal clearance. The tendency of MNPs to accumulate in certain organs will affect their concentration in target sites such cancerous cells and adversely affect the overall therapeutic outcome [122]. It is therefore important to carefully design the NPs to be evenly distributed to the diseased cell or tissues for optimal therapeutic outcome.

## 6. Nanomedicine Research Boom and Clinical Bust: The Uphill Task

The term “nanomedicine” was first used in 1991 in the book of Unbounding the Future by Drexler, Peterson, and Pergamit [123]. After over three decades of research in nanomedicine, one would expect a concomitant boom in the use of the nanotechnologically delivered drugs in the clinics to address the unmet needs with other traditional forms of delivery. More so, it would be expected that so many difficult-to-treat diseases such as cancer and resistant infections would have been expressly overcome. Regrettably, this is not the case as research boom in nanomedicine does not correspond to a boom in the use of technology. Only a few of the drugs have managed to reach clinical stage of use, while many others are still at different phases of development.

Many factors as discussed earlier hinder the express use of nanotechnology for drug delivery. To circumvent some of the challenges such as poor composition, toxicity and biocompatibility, cost and regulatory challenges, paradigm shifts are necessary to make their use come to fruition.

Recently, remarkable improvements have been made in the mechanisms of NP-drug delivery. The improvements in delivery are made by exploiting the peculiarities of each disease condition. For instance, most diseases result in hypoxia and acidosis. Coating NP-drugs with materials that have high affinity for such physiological environments increases targeting. Coating NPs with pH Low Insertion Peptides (pHLIPs) increases efficiency of targeting acidic diseased tissues like cancer cells [124,125]. Furthermore, resistant infections are characterized by modifications in the structure and metabolism, which enable them to survive in the presence of anti-infective agents. NP-drugs are designed to thwart such modifications, to ensure continued efficacy of the drug. For instance, pathogenic bacteria develop quorum sensing (QS), which enables cell-to-cell communication and the formation of virulence factors that increase the severity of the infection [126]. Advancements in NP-drug delivery have targeted the QS to prevent cell-to-cell communication in a process termed quorum quenching (QQ) [127]. Silver nanoparticles have been shown to distort the production of QS and the accompanying virulence in *Pseudomonas aeruginosa*, *Serratia marcescens*, and *Chromobacterium violaceum* [128,129].

Other recent advances include the specific targeting via intravascular and extravascular targeting aimed at reaching diseased locations by NP delivery [130]. The intravascular targeting utilizes ligand binding in the blood vessels, binding peptides, antibodies, and other ligands, coating the surface with cell membranes, and binding the NPs to external physical stimuli [130]. While the surface coating (Biomimetic nano-delivery system) ensures that the NP-drug is not degraded by the immune system, the physical triggering strategy uses ultrasound, magnetic fields, and light for precise targeting [130,131]. Nanotechnology have also been advanced to deliver to the brain by crossing the blood brain barrier (BBB) via extravascular targeting. These afore-discussed advancements in delivery and packaging should be encouraged in the design of NP-drugs in order to reduce toxicity and improve biocompatibility.

The cost of research and development of innovations like NP-drugs is usually huge and is further complicated with the high cost of production. This makes the market price of the final product too high for the masses to afford. This defeats the aim of the innovation as only few can afford them. To ensure that cost of the products is reduced significantly to increase affordability, huge funding is required from donor agencies like Non-Governmental Organizations (NGOs), private firms and Government agencies and parastatals. More so, funding of drug purchase through mandatory health insurance schemes should be encouraged to enable patients to buy such drugs with ease.

To overcome the regulatory hurdles encountered in the registration and use of NP-drugs, the policies on the use of these innovations should be streamlined, and a universal policy developed to ease approval and pharmacovigilance on NP-drugs. A robust global policy will encourage the use of NP-drugs across continents and countries with ease.

Implementation of the expert opinions in this review will go a long way in translating the research boom and the benefits found so far in nanomedicine to boom in the use of the products in clinics.

## 7. Prospects of Nanotechnology in Medicine

Nanotechnology holds a lot of promises in medicine. It will be the future of medicine in predicting, diagnosing and delivering materials for treating diseases. Each of these segments has to be harnessed to obtain the benefits therein.

Nanotechnology has found usefulness in several areas in medicine such as imaging techniques and diagnostic tools, drug delivery systems, tissue-engineered constructs, implants and pharmaceutical therapeutics [132]. These areas of medicine are explored and exploited using nanomedicine to improve healthcare delivery. Recently, some nanoparticles like carbon nanotube, magnetic nanoparticles, quantum dots, and gold NPs have shown the ability to repair damaged cells [133]. This can be exploited in tissue regeneration in degenerative chronic conditions like Alzheimer’s disease. More so, NPs like silver-NPs and Gold-NPs are known to be sun blockers, hence can be used as additives in lotions to act as sun shields [133]. These opportunities presented by nanotechnology in medicine should be exploited via effective collaboration between researchers and the industry.

### 7.1. Tissue Regeneration/Tissue Engineering Construct

Some disease conditions are characterized by loss or malfunctioning of cells, tissues, organs and/or systems. A new approach in medicine targets the regeneration of the worn out tissues in order to restore their functions [134]. This approach is an emerging area of medicine aimed at treating several chronic conditions characterized by loss in cellular functions. This technique is generally called tissue engineering (TE). The use of tissue engineering approaches is limited by several challenges that include, but are not limited to, lack of appropriate biomaterials, ineffective cell growth and absence of techniques to capture the right physiological architectures [135].

The unique properties of each class of NPs make them handy in tissue engineering. These properties exploited in tissue engineering are as outlined in Table 3.

Tissue regeneration involves these main stages: Cell recruitment, growth, proliferation and differentiation. The last stage, differentiation, is a very crucial one for the success of regeneration [136]. Recent studies have shown that NPs enhance differentiation. For instance, in bone TE, it was first observed that gold NPs promote osteogenic differentiation of an osteoblast precursor cell line, MC3T3-E1 [137,138]. Harnessing the potentials of nanotechnology in tissue regeneration can change the fortunes in the management of several chronic conditions like diabetes, Parkinson disease, Alzheimer’s disease and other degenerative diseases.

### 7.2. Potentiation of Immunotherapy

Immunotherapy entails the use of substances that boost or stimulate the immune system to fight diseases. This approach has been used in the treatment of infectious diseases, autoimmune diseases and cancer [139]. The immune system is made up of innate and adaptive immunity, both of which are required to fight diseases. The development of adaptive immunity is the principle behind vaccine development, which is aimed at protecting the masses from infections [140]. Adjuvants enhance the immunogenicity of vaccines to antigens. In vaccine development, NPs encapsulate or adsorb the vaccine antigen or DNA in an appropriate formulation, acting as adjuvants which increase their stability, cellular uptake, and immunogenicity [141]. This new area can be explored effectively to ensure better therapeutic output from vaccines.

Immunotherapy approach is also a necessary paradigm shift in the treatment of cancer as conventional chemotherapy has failed due to resistance development [142]. It becomes imperative to use agents that can stimulate the patients’ immune system against tumors by enhancing the innate immune system and the effector cell number, while, minimizing the host’s suppressor mechanisms [143]. However, many immunotherapies have been hampered by poor delivery and disturbing side effects [143]. The use of NPs in immunotherapy is therefore sought to improve delivery and the therapeutic outcome in this procedure and can be seen as the future of immunotherapy.

### 7.3. Medical Implants

Medical implants are devices or tissues that are carefully kept inside or on the surface of the body with the aim of replacing missing body parts, delivering drugs, diagnosis and/or providing support to organs and tissues. Medical implants have gained much attention in orthopedic medicine; however, the problem of poor osseointegration common with clinically available orthopedic implants limits their use [144] and calls for a paradigm shift. Because NPs act like native cells, they offer better advantages over the conventional approach like better bone fracture repair, promote cell growth, reduce infection rates and biofilm formation [144,145]. More so, some disease conditions cannot be tackled by using orally administered drugs. The use of implantable drugs is the way forward. However, there has to be a balance between safety and efficacy in the drug delivery.

## 8. Conclusions and Future Perspectives

Drugs have been frequently delivered by the traditional means. Poor bioavailability and changes in plasma drug level characterize traditional drug delivery. These traditional means do not allow the optimization of the therapeutic benefits inherent in drugs. A newer approach gives hope for this optimization via improved efficacy and reduced toxicity. To drive home the benefits of nanotechnology in drug delivery, the challenges faced, including cost, ethics and regulatory hurdles must be addressed. This will change the face of medicine in order to attend to the current challenges of drug failure. Increased funding of research and development in NP-drugs, streamlining regulatory hurdles and implementation of the recent advances in nanotechnology are imperative to boost the use of nanotechnology in medicine. Implementation of the expert opinions in this review will go a long way in translating the research boom and the benefits found so far in nanomedicine to boom in the use of the products in clinics.

## Figures and Tables

**Table 3:** Properties of nanomaterials exploited for tissue engineering.

Type of Nanomaterial	Exploited Property
Gold nanoparticles	Surface conjugation and conduction
Silver, Gold and other metallic nanoparticles and metallic oxides	Antimicrobial ability
Quantum dots	Fluorescence ability
Carbon nanotubes	Electromagnetic ability

Adapted from [135].

## References

[1] Satalkar P., Elger B.S., Shaw D.M. (2016). Defining Nano, Nanotechnology and Nanomedicine: Why Should It Matter?. Sci. Eng. Eth..

[2] Wang A.Z., Langer R., Farokhzad O.C. (2012). Nanoparticle Delivery of Cancer Drugs. Annu. Rev. Med..

[3] Ramsden J.J. (2016). Nanotechnology: An Introduction.

[4] Sultana A., Zare M., Thomas V., Kumar T.S.S., Ramakrishna S. (2022). Nano-Based Drug Delivery Systems: Conventional Drug Delivery Routes, Recent Developments and Future Prospects. Med. Drug Discov..

[5] Gupta J., Fatima M.T., Islam Z., Khan R.H., Uversky V.N., Salahuddin P. (2019). Nanoparticle Formulations in the Diagnosis and Therapy of Alzheimer’s Disease. Int. J. Biol. Macromol..

[6] Tiwari G., Tiwari R., Sriwastawa B., Bhati L., Pandey S., Pandey P., Bannerjee S.K. (2012). Drug Delivery Systems: An Updated Review. Int. J. Pharm. Investig..

[7] Tibbitt M.W., Dahlman J.E., Langer R. (2016). Emerging Frontiers in Drug Delivery. J. Am. Chem. Soc..

[8] Adepu S., Ramakrishna S. (2021). Controlled Drug Delivery Systems: Current Status and Future Directions. Molecules.

[9] Bhattacharya S., Rodriques P., Prajapati B., Bhattacharya S., Rodriques P., Prajapati B. (2023). Introductory Chapter: Advanced Drug Delivery Systems. Advanced Drug Delivery Systems.

[10] Vo T.N., Kasper F.K., Mikos A.G. (2012). Strategies for Controlled Delivery of Growth Factors and Cells for Bone Regeneration. Adv. Drug Deliv. Rev..

[11] Fan D., Cao Y., Cao M., Wang Y., Cao Y., Gong T. (2023). Nanomedicine in Cancer Therapy. Signal Transduct. Target. Ther..

[12] Di Stefano A. (2023). Nanotechnology in Targeted Drug Delivery. Int. J. Mol. Sci..

[13] Salahpour Anarjan F. (2019). Active Targeting Drug Delivery Nanocarriers: Ligands. Nano-Struct. Nano-Objects.

[14] Byrne J.D., Betancourt T., Brannon-Peppas L. (2008). Active Targeting Schemes for Nanoparticle Systems in Cancer Therapeutics. Adv. Drug Deliv. Rev..

[15] Attia M.F., Anton N., Wallyn J., Omran Z., Vandamme T.F. (2019). An Overview of Active and Passive Targeting Strategies to Improve the Nanocarriers Efficiency to Tumour Sites. J. Pharm. Pharmacol..

[16] Lee J.H., Yeo Y. (2015). Controlled Drug Release from Pharmaceutical Nanocarriers. Chem. Eng. Sci..

[17] Siepmann J., Siegel R.A., Rathbone M.J. (2012). Fundamentals and Applications of Controlled Release Drug Delivery. Fundamentals and Applications of Controlled Release Drug Delivery.

[18] de Jesus R.A., Oliveira Í.M., Nascimento V.R.S., Ferreira L.F.R., Figueiredo R.T. (2023). Porous Nanostructured Metal Oxides as Potential Scaffolds for Drug Delivery. Novel Platforms for Drug Delivery Applications.

[19] Little T.A. (2014). Toxicological Assessment of Degradation Products: Is It Relevant as a Complementary Approach during Stability Testing of Pharmaceuticals?. J. Dev. Drugs.

[20] Rizvi S.A.A., Saleh A.M. (2018). Applications of Nanoparticle Systems in Drug Delivery Technology. Saudi Pharm. J. SPJ.

[21] Li S.D., Huang L. (2008). Pharmacokinetics and Biodistribution of Nanoparticles. Mol. Pharm..

[22] Niu M., Lu Y., Hovgaard L., Guan P., Tan Y., Lian R., Qi J., Wu W. (2012). Hypoglycemic Activity and Oral Bioavailability of Insulin-Loaded Liposomes Containing Bile Salts in Rats: The Effect of Cholate Type, Particle Size and Administered Dose. Eur. J. Pharm. Biopharm..

[23] Wang N., Wang T., Li T., Deng Y. (2009). Modulation of the Physicochemical State of Interior Agents to Prepare Controlled Release Liposomes. Coll. Surf. B Biointerfaces.

[24] Santos Giuberti C.D., De Oliveira Reis E.C., Ribeiro Rocha T.G., Leite E.A., Lacerda R.G., Ramaldes G.A., De Oliveira M.C. (2011). Study of the Pilot Production Process of Long-Circulating and PH-Sensitive Liposomes Containing Cisplatin. J. Liposome Res..

[25] Afergan E., Epstein H., Dahan R., Koroukhov N., Rohekar K., Danenberg H.D., Golomb G. (2008). Delivery of Serotonin to the Brain by Monocytes Following Phagocytosis of Liposomes. J. Control. Release.

[26] Pandey R., Ahmad Z., Sharma S., Khuller G.K. (2005). Nano-Encapsulation of Azole Antifungals: Potential Applications to Improve Oral Drug Delivery. Int. J. Pharm..

[27] Paavola A., Kilpeläinen I., Yliruusi J., Rosenberg P. (2000). Controlled Release Injectable Liposomal Gel of Ibuprofen for Epidural Analgesia. Int. J. Pharm..

[28] Van Den Hoven J.M., Van Tomme S.R., Metselaar J.M., Nuijen B., Beijnen J.H., Storm G. (2011). Liposomal Drug Formulations in the Treatment of Rheumatoid Arthritis. Mol. Pharm..

[29] Marques J., Moles E., Urbán P., Prohens R., Busquets M.A., Sevrin C., Grandfils C., Fernàndez-Busquets X. (2014). Application of Heparin as a Dual Agent with Antimalarial and Liposome Targeting Activities toward Plasmodium-Infected Red Blood Cells. Nanomedicine.

[30] Yu J.Y., Chuesiang P., Shin G.H., Park H.J. (2021). Post-Processing Techniques for the Improvement of Liposome Stability. Pharmaceutics.

[31] Lister J. (1996). Amphotericin B Lipid Complex (Abelcet) in the Treatment of Invasive Mycoses: The North American Experience. Eur. J. Haematol. Suppl..

[32] Blair H.A. (2018). Daunorubicin/Cytarabine Liposome: A Review in Acute Myeloid Leukaemia. Drugs.

[33] Smith J.A., Mathew L., Burney M., Nyshadham P., Coleman R.L. (2016). Equivalency Challenge: Evaluation of Lipodox® as the Generic Equivalent for Doxil® in a Human Ovarian Cancer Orthotropic Mouse Model. Gynecol. Oncol..

[34] Keam S.J., Scott L.J., Curran M.P. (2003). Verteporfin: A Review of Its Use in the Management of Subfoveal Choroidal Neovascularisation. Drugs.

[35] Jaeckle K.A., Phuphanich S., Van den Bent M.J., Aiken R., Batchelor T., Campbell T., Fulton D., Gilbert M., Heros D., Rogers L. (2001). Intrathecal Treatment of Neoplastic Meningitis Due to Breast Cancer with a Slow-Release Formulation of Cytarabine. Br. J. Cancer.

[36] Hartrick C.T., Hartrick K.A. (2008). Extended-Release Epidural Morphine (DepoDur): Review and Safety Analysis. Expert Rev. Neurother..

[37] Gregoriadis G. (2021). Liposomes and MRNA: Two Technologies Together Create a COVID-19 Vaccine. Med. Drug Discov..

[38] Kager L., Pötschger U., Bielack S. (2010). Review of Mifamurtide in the Treatment of Patients with Osteosarcoma. Ther. Clin. Risk Manag..

[39] Singh G., Song S., Choi E., Lee P.B., Nahm F.S. (2020). Recombinant Zoster Vaccine (Shingrix^®^): A New Option for the Prevention of Herpes Zoster and Postherpetic Neuralgia. Korean J. Pain.

[40] Sanofi Aventis TAXOTERE® (Docetaxel) Injection, for Intravenous Use. https://products.sanofi.us/taxotere/taxotere.html.

[41] Novavax (2003). Estradiol-Topical--Novavax: Estrasorb. Drugs R D..

[42] Pellett Madan R., Dezzutti C.S., Rabe L., Hillier S.L., Marrazzo J., Mcgowan I., Richardson B.A., Herold B.C. (2015). Soluble Immune Mediators and Vaginal Bacteria Impact Innate Genital Mucosal Antimicrobial Activity in Young Women. Am. J. Reprod. Immunol..

[43] Ju J., Rh M. (2008). Nanocrystal Technology, Drug Delivery and Clinical Applications. Int. J. Nanomed..

[44] Caster J.M., Patel A.N., Zhang T., Wang A. (2017). Investigational Nanomedicines in 2016: A Review of Nanotherapeutics Currently Undergoing Clinical Trials. Wiley Interdiscip. Rev. Nanomed. Nanobiotechnol..

[45] Deschamps B., Musaji N., Gillespie J.A. (2009). Food Effect on the Bioavailability of Two Distinct Formulations of Megestrol Acetate Oral Suspension. Int. J. Nanomed..

[46] Chavez B., Sopko M.A., Ehret M.J., Paulino R.E., Goldberg K.R., Angstadt K., Bogart G.T. (2009). An Update on Central Nervous System Stimulant Formulations in Children and Adolescents with Attention-Deficit/Hyperactivity Disorder. Ann. Pharmacother..

[47] Kaddar N., Vigneault P., Pilote S., Patoine D., Simard C., Drolet B. (2012). Tizanidine (Zanaflex): A Muscle Relaxant That May Prolong the QT Interval by Blocking I Kr. J. Cardiovasc. Pharmacol. Ther..

[48] Abdellatif A.A.H., Alsowinea A.F. (2021). Approved and Marketed Nanoparticles for Disease Targeting and Applications in COVID-19. Nanotechnol. Rev..

[49] Ardestani M.S., Zaheri Z., Mohammadzadeh P., Bitarafan-Rajabi A., Ghoreishi S.M. (2021). Novel Manganese Carbon Quantum Dots as a Nano-Probe: Facile Synthesis, Characterization and Their Application in Naproxen Delivery (Mn/CQD/SiO_2_@naproxen). Bioorg. Chem..

[50] Fan Y., Zhang Q. (2013). Development of Liposomal Formulations: From Concept to Clinical Investigations. Asian J. Pharm. Sci..

[51] Fasol U., Frost A., Büchert M., Arends J., Fiedler U., Scharr D., Scheuenpflug J., Mross K. (2012). Vascular and Pharmacokinetic Effects of EndoTAG-1 in Patients with Advanced Cancer and Liver Metastasis. Ann. Oncol..

[52] Halwani A.A. (2022). Development of Pharmaceutical Nanomedicines: From the Bench to the Market. Pharmaceutics.

[53] Thapa R.K., Kim J.O. (2022). Nanomedicine-Based Commercial Formulations: Current Developments and Future Prospects. J. Pharm. Investig..

[54] Zhang Y., Huang Y., Li S. (2014). Polymeric Micelles: Nanocarriers for Cancer-Targeted Drug Delivery. AAPS PharmSciTech.

[55] Ahmad Z., Shah A., Siddiq M., Kraatz H.B. (2014). Polymeric Micelles as Drug Delivery Vehicles. RSC Adv..

[56] Perumal S., Atchudan R., Lee W. (2022). A Review of Polymeric Micelles and Their Applications. Polymers.

[57] Lu Y., Zhang E., Yang J., Cao Z. (2018). Strategies to Improve Micelle Stability for Drug Delivery. Nano Res..

[58] Buhleier E., Wehner E., Vögtle F. (1978). Cascade-Chain like and Nonskid-Chain-like Synthesis of Molecular Cavity Topologies. Synthesis.

[59] Noriega-Luna B., Godínez L.A., Rodríguez F.J., Rodríguez A., Zaldívar-Lelo De Larrea G., Sosa-Ferreyra C.F., Mercado-Curiel R.F., Manríquez J., Bustos E. (2014). Applications of Dendrimers in Drug Delivery Agents, Diagnosis, Therapy, and Detection. J. Nanomater..

[60] Mittal P., Saharan A., Verma R., Altalbawy F.M.A., Alfaidi M.A., Batiha G.E.S., Akter W., Gautam R.K., Uddin M.S., Rahman M.S. (2021). Dendrimers: A New Race of Pharmaceutical Nanocarriers. BioMed Res. Int..

[61] Rai D.B., Pooja D., Kulhari H. (2020). Dendrimers in Gene Delivery. Pharmaceutical Applications of Dendrimers.

[62] Dufès C., Uchegbu I.F., Schätzlein A.G. (2005). Dendrimers in Gene Delivery. Adv. Drug Deliv. Rev..

[63] Farias E.D., Bouchet L.M., Brunetti V., Strumia M.C. (2017). Dendrimers and Dendronized Materials as Nanocarriers. Nanostructures for Novel Therapy.

[64] Ciolkowski M., Petersen J.F., Ficker M., Janaszewska A., Christensen J.B., Klajnert B., Bryszewska M. (2012). Surface Modification of PAMAM Dendrimer Improves Its Biocompatibility. Nanomedicine.

[65] Aurelia Chis A., Dobrea C., Morgovan C., Arseniu A.M., Rus L.L., Butuca A., Juncan A.M., Totan M., Vonica-Tincu A.L., Cormos G. (2020). Applications and Limitations of Dendrimers in Biomedicine. Molecules.

[66] Matea C.T., Mocan T., Tabaran F., Pop T., Mosteanu O., Puia C., Iancu C., Mocan L. (2017). Quantum Dots in Imaging, Drug Delivery and Sensor Applications. Int. J. Nanomed..

[67] Bruno J.G. (2022). Advantages and Disadvantages of Using Quantum Dots in Lateral Flow and Other Biological Assay Formats. Application of Quantum Dots in Biology and Medicine: Recent Advances.

[68] Mitchell M.J., Billingsley M.M., Haley R.M., Wechsler M.E., Peppas N.A., Langer R. (2020). Engineering Precision Nanoparticles for Drug Delivery. Nat. Rev. Drug Discov..

[69] Cai X., Jia H., Liu Z., Hou B., Luo C., Feng Z., Li W., Liu J. (2008). Polyhydroxylated Fullerene Derivative C_60_(OH)_24_ Prevents Mitochondrial Dysfunction and Oxidative Damage in an MPP^+^-Induced Cellular Model of Parkinson’s Disease. J. Neurosci. Res..

[70] Jha R., Singh A., Sharma P.K., Fuloria N.K. (2020). Smart Carbon Nanotubes for Drug Delivery System: A Comprehensive Study. J. Drug Deliv. Sci. Technol..

[71] Nayfeh M. (2018). Toxicity and Safety Issues of Metal-Based Nanoparticles. Fundamentals and Applications of Nano Silicon in Plasmonics and Fullerines: Current and Future Trends.

[72] Yaqoob A.A., Ahmad H., Parveen T., Ahmad A., Oves M., Ismail I.M.I., Qari H.A., Umar K., Mohamad Ibrahim M.N. (2020). Recent Advances in Metal Decorated Nanomaterials and Their Various Biological Applications: A Review. Front. Chem..

[73] Arora N., Thangavelu K., Karanikolos G.N. (2020). Bimetallic Nanoparticles for Antimicrobial Applications. Front. Chem..

[74] Dadfar S.M., Roemhild K., Drude N.I., von Stillfried S., Knüchel R., Kiessling F., Lammers T. (2019). Iron Oxide Nanoparticles: Diagnostic, Therapeutic and Theranostic Applications. Adv. Drug Deliv. Rev..

[75] Bajpai A., Shinde S., Basu S. (2022). Nanobiomaterials for Drug Delivery and Theranostics. Nanotechnology in Medicine and Biology.

[76] Sánchez-López E., Gomes D., Esteruelas G., Bonilla L., Lopez-Machado A.L., Galindo R., Cano A., Espina M., Ettcheto M., Camins A. (2020). Metal-Based Nanoparticles as Antimicrobial Agents: An Overview. Nanomaterials.

[77] Zare H., Ahmadi S., Ghasemi A., Ghanbari M., Rabiee N., Bagherzadeh M., Karimi M., Webster T.J., Hamblin M.R., Mostafavi E. (2021). Carbon Nanotubes: Smart Drug/Gene Delivery Carriers. Int. J. Nanomed..

[78] Wicht K.J., Mok S., Fidock D.A. (2020). Molecular Mechanisms of Drug Resistance in Plasmodium Falciparum Malaria. Annu. Rev. Microbiol..

[79] WHO (2021). World Malaria Report. https://www.who.int/publications/i/item/9789240040496.

[80] Chaves J.B., Portugal Tavares de Moraes B., Regina Ferrarini S., Noé da Fonseca F., Silva A.R., Gonçalves-de-Albuquerque C.F. (2022). Potential of Nanoformulations in Malaria Treatment. Front. Pharmacol..

[81] Tsamesidis I., Lymperaki E., Egwu C.O., Pouroutzidou G.K., Kazeli K., Reybier K., Bourgeade-Delmas S., Valentin A., Kontonasaki E. (2021). Effect of Silica Based Nanoparticles against Plasmodium Falciparum and Leishmania Infantum Parasites. J. Xenobiotics.

[82] Aderibigbe B.A. (2017). Metal-Based Nanoparticles for the Treatment of Infectious Diseases. Mol. A J. Synth. Chem. Nat. Prod. Chem..

[83] Borah Slater K., Kim D., Chand P., Xu Y., Shaikh H., Undale V. (2023). A Current Perspective on the Potential of Nanomedicine for Anti-Tuberculosis Therapy. Trop. Med. Infect. Dis..

[84] Singh R., Smitha M.S., Singh S.P. (2014). The Role of Nanotechnology in Combating Multi-Drug Resistant Bacteria. J. Nanosci. Nanotechnol..

[85] Hetta H.F., Ramadan Y.N., Al-Harbi A.I., Ahmed E.A., Battah B., Abd Ellah N.H., Zanetti S., Donadu M.G. (2023). Nanotechnology as a Promising Approach to Combat Multidrug Resistant Bacteria: A Comprehensive Review and Future Perspectives. Biomedicines.

[86] Aabed K., Mohammed A.E. (2021). Synergistic and Antagonistic Effects of Biogenic Silver Nanoparticles in Combination with Antibiotics Against Some Pathogenic Microbes. Front. Bioeng. Biotechnol..

[87] Adeniji O.O., Ojemaye M.O., Okoh A.I. (2022). Antibacterial Activity of Metallic Nanoparticles against Multidrug-Resistant Pathogens Isolated from Environmental Samples: Nanoparticles/Antibiotic Combination Therapy and Cytotoxicity Study. ACS Appl. Bio Mater..

[88] Zhang Y., Yuan Y., Chen W., Fan J., Lv H., Wu Q. (2019). Integrated Nanotechnology of Synergism-Sterilization and Removing-Residues for Neomycin through Nano-Cu_2_O. Coll. Surfaces B Biointerfaces.

[89] WHO Cancer. https://www.who.int/news-room/fact-sheets/detail/cancer.

[90] Jin C., Wang K., Oppong-Gyebi A., Hu J. (2020). Application of Nanotechnology in Cancer Diagnosis and Therapy—A Mini-Review. Int. J. Med. Sci..

[91] Bukowski K., Kciuk M., Kontek R. (2020). Mechanisms of Multidrug Resistance in Cancer Chemotherapy. Int. J. Mol. Sci..

[92] Cao L., Zhu Y., Wang W., Wang G., Zhang S., Cheng H. (2021). Emerging Nano-Based Strategies Against Drug Resistance in Tumor Chemotherapy. Front. Bioeng. Biotechnol..

[93] Yao Y., Zhou Y., Liu L., Xu Y., Chen Q., Wang Y., Wu S., Deng Y., Zhang J., Shao A. (2020). Nanoparticle-Based Drug Delivery in Cancer Therapy and Its Role in Overcoming Drug Resistance. Front. Mol. Biosci..

[94] He J., Gong C., Qin J., Li M., Huang S. (2019). Cancer Cell Membrane Decorated Silica Nanoparticle Loaded with MiR495 and Doxorubicin to Overcome Drug Resistance for Effective Lung Cancer Therapy. Nanoscale Res. Lett..

[95] Babu A., Munshi A., Ramesh R. (2017). Combinatorial Therapeutic Approaches with RNAi and Anticancer Drugs Using Nanodrug Delivery Systems. Drug Dev. Ind. Pharm..

[96] Bai F., Wang C., Lu Q., Zhao M., Ban F.Q., Yu D.H., Guan Y.Y., Luan X., Liu Y.R., Chen H.Z. (2013). Nanoparticle-Mediated Drug Delivery to Tumor Neovasculature to Combat P-Gp Expressing Multidrug Resistant Cancer. Biomaterials.

[97] Venishetty V.K., Chede R., Komuravelli R., Adepu L., Sistla R., Diwan P.V. (2012). Design and Evaluation of Polymer Coated Carvedilol Loaded Solid Lipid Nanoparticles to Improve the Oral Bioavailability: A Novel Strategy to Avoid Intraduodenal Administration. Coll. Surf. B Biointerfaces.

[98] Kumar V.V., Chandrasekar D., Ramakrishna S., Kishan V., Rao Y.M., Diwan P.V. (2007). Development and Evaluation of Nitrendipine Loaded Solid Lipid Nanoparticles: Influence of Wax and Glyceride Lipids on Plasma Pharmacokinetics. Int. J. Pharm..

[99] Caponio G.R., Lippolis T., Tutino V., Gigante I., De Nunzio V., Milella R.A., Gasparro M., Notarnicola M. (2022). Nutraceuticals: Focus on Anti-Inflammatory, Anti-Cancer, Antioxidant Properties in Gastrointestinal Tract. Antioxidants.

[100] Parada J., Aguilera J.M. (2007). Food Microstructure Affects the Bioavailability of Several Nutrients. J. Food Sci..

[101] Manocha S., Dhiman S., Grewal S.A., Guarve K. (2022). Nanotechnology: An Approach to Overcome Bioavailability Challenges of Nutraceuticals. J. Drug Deliv. Sci. Technol..

[102] Shin G.H., Kim J.T., Park H.J. (2015). Recent Developments in Nanoformulations of Lipophilic Functional Foods. Trends Food Sci. Technol..

[103] Dima C., Assadpour E., Dima S., Jafari S.M. (2020). Bioavailability of Nutraceuticals: Role of the Food Matrix, Processing Conditions, the Gastrointestinal Tract, and Nanodelivery Systems. Compr. Rev. Food Sci. Food Saf..

[104] Herranz F., Almarza E., Rodríguez I., Salinas B., Rosell Y., Desco M., Bulte J.W., Ruiz-Cabello J. (2011). The Application of Nanoparticles in Gene Therapy and Magnetic Resonance Imaging. Microsc. Res. Tech..

[105] Mirza Z., Karim S. (2021). Nanoparticles-Based Drug Delivery and Gene Therapy for Breast Cancer: Recent Advancements and Future Challenges. Semin. Cancer Biol..

[106] Piperno A., Sciortino M.T., Giusto E., Montesi M., Panseri S., Scala A. (2021). Recent Advances and Challenges in Gene Delivery Mediated by Polyester-Based Nanoparticles. Int. J. Nanomed..

[107] Xu Z., Wang D., Cheng Y., Yang M., Wu L.P. (2018). Polyester Based Nanovehicles for SiRNA Delivery. Mater. Sci. Eng. C.

[108] Prazeres P.H.D.M., Ferreira H., Costa P.A.C., da Silva W., Alves M.T., Padilla M., Thatte A., Santos A.K., Lobo A.O., Sabino A. (2023). Delivery of Plasmid DNA by Ionizable Lipid Nanoparticles to Induce CAR Expression in T Cells. Int. J. Nanomed..

[109] Hou X., Zaks T., Langer R., Dong Y. (2021). Lipid Nanoparticles for MRNA Delivery. Nat. Rev. Mater..

[110] Desai N. (2012). Challenges in Development of Nanoparticle-Based Therapeutics. AAPS J..

[111] Sharma S., Parveen R., Chatterji B.P. (2021). Toxicology of Nanoparticles in Drug Delivery. Curr. Pathobiol. Rep..

[112] Li X., Wang L., Fan Y., Feng Q., Cui F.Z. (2012). Biocompatibility and Toxicity of Nanoparticles and Nanotubes. J. Nanomater..

[113] Pandey G., Jain P. (2020). Assessing the Nanotechnology on the Grounds of Costs, Benefits, and Risks. Beni-Suef Univ. J. Basic Appl. Sci..

[114] Allan J., Belz S., Hoeveler A., Hugas M., Okuda H., Patri A., Rauscher H., Silva P., Slikker W., Sokull-Kluettgen B. (2021). Regulatory Landscape of Nanotechnology and Nanoplastics from a Global Perspective. Regul. Toxicol. Pharmacol..

[115] Pita R., Ehmann F., Papaluca M. (2016). Nanomedicines in the EU-Regulatory Overview. AAPS J..

[116] Fay F., Hansen L., Hectors S.J.C.G., Sanchez-Gaytan B.L., Zhao Y., Tang J., Munitz J., Alaarg A., Braza M.S., Gianella A. (2017). Investigating the Cellular Specificity in Tumors of a Surface-Converting Nanoparticle by Multimodal Imaging. Bioconjug. Chem..

[117] Mukherjee B., Dey N.S., Maji R., Bhowmik P., Das P.J., Paul P. (2014). Current Status and Future Scope for Nanomaterials in Drug Delivery. Application of Nanotechnology in Drug Delivery.

[118] Singh R., Lillard J.W. (2009). Nanoparticle-Based Targeted Drug Delivery. Exp. Mol. Pathol..

[119] Hainfeld J.F., Lin L., Slatkin D.N., Avraham Dilmanian F., Vadas T.M., Smilowitz H.M. (2014). Gold Nanoparticle Hyperthermia Reduces Radiotherapy Dose. Nanomedicine Nanotechnol. Biol. Med..

[120] Poller W.C., Löwa N., Wiekhorst F., Taupitz M., Wagner S., Möller K., Baumann G., Stangl V., Trahms L., Ludwig A. (2016). Magnetic Particle Spectroscopy Reveals Dynamic Changes in the Magnetic Behavior of Very Small Superparamagnetic Iron Oxide Nanoparticles During Cellular Uptake and Enables Determination of Cell-Labeling Efficacy. J. Biomed. Nanotechnol..

[121] De Jong W.H., Hagens W.I., Krystek P., Burger M.C., Sips A.J.A.M., Geertsma R.E. (2008). Particle Size-Dependent Organ Distribution of Gold Nanoparticles after Intravenous Administration. Biomaterials.

[122] Waite C.L., Roth C.M. (2012). Nanoscale Drug Delivery Systems for Enhanced Drug Penetration into Solid Tumors: Current Progress and Opportunities. Crit. Rev. Biomed. Eng..

[123] Drexler K.E., Peterson C., Pergamit G. (1991). Unbounding the Future: The Nanotechnology Revolution.

[124] Arachchige M.C.M., Reshetnyak Y.K., Andreev O.A. (2015). Advanced Targeted Nanomedicine. J. Biotechnol..

[125] Ibrahim-Hashim A., Estrella V. (2019). Acidosis and Cancer: From Mechanism to Neutralization. Cancer Metastasis Rev..

[126] Maruthupandy M., Rajivgandhi G.N., Quero F., Li W.J. (2020). Anti-Quorum Sensing and Anti-Biofilm Activity of Nickel Oxide Nanoparticles against *Pseudomonas aeruginosa*. J. Environ. Chem. Eng..

[127] Ozdal M., Gurkok S. (2022). Recent Advances in Nanoparticles as Antibacterial Agent. ADMET DMPK.

[128] Coman A.N., Mare A., Tanase C., Bud E., Rusu A. (2021). Silver-Deposited Nanoparticles on the Titanium Nanotubes Surface as a Promising Antibacterial Material into Implants. Metals.

[129] Qais F.A., Shafiq A., Ahmad I., Husain F.M., Khan R.A., Hassan I. (2020). Green Synthesis of Silver Nanoparticles Using Carum Copticum: Assessment of Its Quorum Sensing and Biofilm Inhibitory Potential against Gram Negative Bacterial Pathogens. Microb. Pathog..

[130] Cheng X., Xie Q., Sun Y. (2023). Advances in Nanomaterial-Based Targeted Drug Delivery Systems. Front. Bioeng. Biotechnol..

[131] Chen M., Chen M., He J. (2019). Cancer Cell Membrane Cloaking Nanoparticles for Targeted Co-Delivery of Doxorubicin and PD-L1 SiRNA. Artif. Cells Nanomed. Biotechnol..

[132] Sim S., Wong N.K. (2021). Nanotechnology and Its Use in Imaging and Drug Delivery (Review). Biomed. Rep..

[133] Kanaoujiya R., Saroj S.K., Rajput V.D., Alimuddin, Srivastava S., Minkina T., Igwegbe C.A., Singh M., Kumar A. (2023). Emerging Application of Nanotechnology for Mankind. Emergent Mater..

[134] Whitney G.A., Jayaraman K., Dennis J.E., Mansour J.M. (2017). Scaffold-Free Cartilage Subjected to Frictional Shear Stress Demonstrates Damage by Cracking and Surface Peeling. J. Tissue Eng. Regen. Med..

[135] Hasan A., Morshed M., Memic A., Hassan S., Webster T.J., Marei H.E.S. (2018). Nanoparticles in Tissue Engineering: Applications, Challenges and Prospects. Int. J. Nanomed..

[136] Santos A.R., Nascimento V.A., Genari S.C., Lombello C.B., Nascimento V.A., Genari S.C., Lombello C.B. (2014). Mechanisms of Cell Regeneration—From Differentiation to Maintenance of Cell Phenotype. Cells Biomater. Regen. Med..

[137] Boisselier E., Astruc D. (2009). Gold Nanoparticles in Nanomedicine: Preparations, Imaging, Diagnostics, Therapies and Toxicity. Chem. Soc. Rev..

[138] Chen Y., Li N., Yang Y., Liu Y. (2015). A Dual Targeting Cyclodextrin/Gold Nanoparticle Conjugate as a Scaffold for Solubilization and Delivery of Paclitaxel. RSC Adv..

[139] Qadri H., Shah A.H., Alkhanani M., Almilaibary A., Mir M.A. (2023). Immunotherapies against Human Bacterial and Fungal Infectious Diseases: A Review. Front. Med..

[140] Kang S.M., Compans R.W. (2009). Host Responses from Innate to Adaptive Immunity after Vaccination: Molecular and Cellular Events. Mol. Cells.

[141] Coffman R.L., Sher A., Seder R.A. (2010). Vaccine Adjuvants: Putting Innate Immunity to Work. Immunity.

[142] Alfarouk K.O., Stock C.M., Taylor S., Walsh M., Muddathir A.K., Verduzco D., Bashir A.H.H., Mohammed O.Y., Elhassan G.O., Harguindey S. (2015). Resistance to Cancer Chemotherapy: Failure in Drug Response from ADME to P-Gp. Cancer Cell Int..

[143] Debele T.A., Yeh C.F., Su W.P. (2020). Cancer Immunotherapy and Application of Nanoparticles in Cancers Immunotherapy as the Delivery of Immunotherapeutic Agents and as the Immunomodulators. Cancers.

[144] Pokkalath A., Nadar D., Ravikumar P., Sawarkar S.P. (2021). Nanomaterials for Orthopaedic Implants and Applications. Handbook on Nanobiomaterials for Therapeutics and Diagnostic Applications.

[145] Tomsia A.P., Launey M.E., Lee J.S., Mankani M.H., Wegst U.G.K., Saiz E. (2011). Nanotechnology Approaches for Better Dental Implants. Int. J. Oral Maxillofac. Implants.

